# The Use of Wearable Activity Trackers in Schools to Promote Child and Adolescent Physical Activity: A Descriptive Content Analysis of School Staff’s Perspectives

**DOI:** 10.3390/ijerph192114067

**Published:** 2022-10-28

**Authors:** Amy V. Creaser, Marie T. Frazer, Silvia Costa, Daniel D. Bingham, Stacy A. Clemes

**Affiliations:** 1 School of Sport, Exercise, and Health Sciences, Loughborough University, Loughborough LE11 3TU, UK; 2 Bradford Institute for Health Research, Bradford Teaching Hospitals Foundation Trust, Bradford BD9 6RJ, UK; 3 Faculty of Health Studies, University of Bradford, Richmond Road, Bradford BD7 1DP, UK; 4 National Institute for Health Research (NIHR) Leicester Biomedical Research Centre, University Hospitals of Leicester NHS Trust, University of Leicester, Leicester LE5 4PW, UK

**Keywords:** school, wearable activity trackers, physical activity, children, adolescents

## Abstract

Background: The school environment is an ideal setting for promoting physical activity (PA). Wearable activity trackers (wearables) have previously been implemented, in research, as intervention tools within the school-environment. However, the large-scale use and acceptance of wearables, in schools, is unknown. Methods: This study distributed a cross-sectional survey to school staff to investigate the prevalence of child and adolescent wearable use in schools, including when and how they are used, and school staff’s willingness to use them in the future (as implemented by school staff). This survey consisted of between 13 and 22 items, including closed-ended and open-ended questions. Closed-ended responses were displayed descriptively (wearable prevalence and characteristics), and open-ended qualitative responses were categorised using descriptive content analysis (how wearables are used). Results: 1087 school staff provided valid responses. Of those, 896 (82.4%) had never used a wearable as a teaching or support tool for their students, and 120 (11%) currently used- and 71 (6.5%) had previously used- a wearable as a teaching or support tool for their students. When wearables were used, school staff implemented their use regularly and during physical education lessons or throughout the entire school day. Wearables were used to monitor or increase student’s PA levels, or for student and staff educational purposes (e.g., academic learning, movement breaks). Most school staff were willing to use a wearable as a teaching or support tool to promote student’s PA, and/or learning about PA, in the future. Conclusions: This study is the first study to explore the widescale use and acceptance of children and adolescents using wearables in the school-setting. Findings may inform the development of future school-based interventions and public health initiatives for physical activity promotion, using wearables.

## 1. Introduction

Physical activity (PA) has been associated with cognitive function and academic achievement, in children and adolescents [1]. However, PA levels during school hours are generally low [2,3]. Approximately 83% of a child’s school day is spent in academic lessons, which also happens to be the least active part of the school day [3]. Previous research has found that children (aged 9- to 10-years) participate in an average of 7.81 min of moderate-to-vigorous-intensity PA (MVPA) during academic lessons [3]. Despite this, schools have been coined as an ideal setting for promoting PA in children and adolescents, given the amount of time young people spend in school, and the availability of school’s resources which may enable implementation (e.g., staff and facilities) [4]. There is growing interest therefore in how interventions to promote PA in children/adolescents can be implemented within the school environment.

The effectiveness of school-based PA interventions are largely mixed [5], however there is some evidence that school-based interventions incorporating goal setting, action planning, feedback and self-monitoring can increase children’s/adolescent’s PA [6]. Wearable activity trackers (wearables) include features that correspond with these behaviour change techniques [7]. Wearables, such as Fitbit and Garmin devices, go beyond a traditional step-only display by tracking multiple dimensions of PA, including other gamification features, and enabling short- and long-term monitoring using a monitoring display and access to an app [8,9]. A recent systematic review exploring the acceptability, feasibility and effectiveness of wearables on child and adolescent PA found that 52% (*n* = 17/33) of studies utilised wearables in a school-setting (versus other settings) [8]. There is some preliminary evidence that school-based wearable interventions can increase step counts and time in MVPA [10,11,12]. However, it is important to consider the habitual use and acceptability of using wearables in schools, given school staff have previously reported numerous barriers to implementing PA interventions and policies, such as lack of time and space, financial constraints [13] and prioritising traditional academic subjects [14].

Few studies have investigated teacher perspectives of using wearables in schools to promote child or adolescent health, well-being or learning [15,16]. Of those that have, most explore physical education (P.E.) teachers perspectives of integrating wearables into P.E. lessons were explored [15,16]. These studies reported that wearables could be easily integrated into P.E. lessons, and enabled P.E. teachers to monitor and promote their students’ PA [15,16]. However, several barriers were identified, including lack of additional technology to support wearable use (e.g., computers/laptops to sync devices), risk of injury, and a lack of school funding to purchase additional devices [15,16]. These findings highlight the key benefits and drawbacks of using wearables within P.E. lessons. However, the perspectives of wearable use to promote PA within schools from all school staff, such as teachers of classroom-based subjects, corresponding to the most inactive periods of the school day, have had little examination. The World Health Organisation’s policy brief for ‘promoting PA through schools’ recognises the importance of creating active classrooms alongside P.E. lessons and opportunities for PA during recess, within the school day [17]. Suggestions for creating active classrooms include incorporating short (3–5 min) active movement breaks and PA into the delivery of academic content (e.g., counting steps walked to calculate distance) into academic lessons or restructuring the classroom environment (e.g., standing desks) [17]. Incorporating PA into academic lessons, in addition to P.E. lessons and recess periods, can increase total daily PA levels in 3- to 14-year-olds [18]. Therefore, gathering the perspectives of any school staff member, including classroom teachers, P.E. teachers, midday staff (supervising recess and lunch periods) and leadership staff, can provide an insight into how wearables are used, or can be used in the future, in schools to promote child PA levels across the whole school day.

A previous study has found that classroom teachers express willingness to use wearables to measure and monitor their student’s PA levels [19]. However, this study did not explore the habitual widescale use of wearables, and how school staff use or would be willing to use them in a typical school day. Understanding this will provide insights into how wearables can be implemented into schools to increase child and adolescent PA levels, at a population-level, and inform future school-based interventions or policies.

This exploratory study used a descriptive content analysis to explore responses to a cross-sectional online survey to address the following aims: (1) to examine the prevalence of child and adolescent wearable use in schools (as implemented by school staff), and to understand how and when school staff use wearables in schools with their students, (2) to examine school staff’s reasons for using and not using wearables to teach or support their students, and (3) to determine school staff’s willingness to implement the use of wearables with their students to promote child and adolescent PA in schools, in the future (non-users or previous users), including when and how.

## 2. Methods

### 2.1. Design

This study utilised a cross-sectional survey to investigate the prevalence and use of wearables in schools by children and adolescents, as implemented by school staff. This study received ethical approval from Loughborough University Ethical Approvals (Human Participants) Sub-Committee (REF 2021-5092-3976).

### 2.2. Participants

School staff members (e.g., classroom teachers, headteachers, P.E. teachers, support staff) who worked with students aged 5- to 16-years-old were invited to complete an online survey. School staff working in any country could complete the survey.

### 2.3. Procedure

The survey was hosted by ‘Online Surveys’ (https://www.onlinesurveys.ac.uk/, accessed on 10 March 2021). The survey was advertised on social media (e.g., Twitter and Facebook teacher forums, that encourage discussions between school staff working across different age groups, subjects, and roles). Questions and answers were written in the English language. Before the survey could be completed, eligibility was assessed (“Are you a member of staff (aged 18 years or above) at a Primary or Secondary school, working with pupils/students aged 5 to 16 years?”). Eligible participants were then provided with an information sheet within the online survey platform, and consent was provided by selecting a check box.

Prior to the survey questions, the following definitions were outlined, to provide clarity throughout the survey:

- Wearable activity/fitness tracker(s)—“This refers to devices that can be worn on the body (most commonly on the wrist like a watch) that measures how much physical activity you have done. This may include how many steps, or miles you have walked. Some wearable activity/fitness trackers also measure how quickly your heart is beating, how much you sleep, and may remind you to be active. They include devices such as Fitbit, Garmin, Misfit, Apple iWatch, or other similar devices”,
- Teaching/support tool—“This refers to the pupils/students you work with using the wearable activity/fitness tracker as part of a typical school day/week/month/year or as part of the school curriculum. E.g., students wearing them in P.E./during break times so you can monitor their physical activity, wearing them in class to teach them mathematics or encourage them to be physically active)”.

All respondents were entered into a prize draw to win one of three £20 online shopping vouchers. The complete survey took approximately 20-min to complete and was available between May and August 2021 (68 days).

### 2.4. Measures

Supplementary Table S1 displays the survey questions, and their corresponding response options. The survey consisted of between 13 and 22 items (including demographic questions), depending on whether the staff member; (1) currently (*n* = 18), (2) previously (but no longer) (*n* = 22), or (3) had never (*n* = 13) implemented the use of wearables as a teaching/support tool within a school. Most questions were closed-ended, with two being open-ended (how wearables are used (currently use and previously used) and how would school staff be willing to use wearables in the future to support child or adolescent health, well-being, or learning (previously and never used)).

### 2.5. Data Analysis

The number and frequency (*n*, %) of closed-question responses are presented. Responses between school staff who worked in primary schools (5- to 11-years) and secondary schools (11- to 16-years) were initially explored to identify any differences in responses (based on the UK’s educational system [20]). There were no apparent differences in responses, therefore results were combined.

#### Content Analysis

For open-ended qualitative responses, a descriptive content analysis, using the guidance outlined by Bengtsson [21], was conducted. This addressed the research aims one and three: how wearables are used, and how school staff would be willing to use wearables, in school to promote PA. Previous research has used content analysis to summarise survey responses exploring young adult’s social media use, addressing similar aims to the current study (what is used, why and how) [22]. Two authors (AVC and MTF) conducted the content analysis. AVC familiarised themselves with the data and inductively developed meaning units from the data (decontextualisation). Meaning units contain insights into the data and consider how the data aligns with the study’s research aims [21]. This involved AVC considering how answers reflected how wearables were used in schools. These meaning units were then ‘recontextualised’ by MTF, who checked whether the units reflected all aspects of the data, and accurately addressed the research aims (e.g., do they reflect how wearables are being used versus why or when they are used, etc.) [21]. Once the meaning units were contextualised, AVC independently developed the final meaning units into overarching themes and sub-categories [21]. Categories and sub-categories were discussed with MTF, who provided critical insights into whether categories could be refined, subdivided or other categories added. Once categories were determined, AVC and MTF independently used an online spreadsheet which included each respondent’s open-ended response (Y axis) and each sub-category (X-axis). Authors familiarised themselves with each response and indicated which category the response reflected (Yes/No). Responses could reflect more than one category. Initially, 200 responses were coded independently, with any disagreements discussed and resolved. Once it had been determined that AVC and MTF were following the same procedure and in agreement with the results, the remaining responses were similarly coded, with any disagreements also resolved. From this, the number (*n*, %) of responses in each category were calculated.

## 3. Results

A total of 1119 school staff members completed the survey. Responses from 32 participants were removed, as responses indicated personal wearable use with no application to use within the school environment (e.g., did not involve the students using the wearable; “I don’t relate my activity to the pupils’ activity”, “track how many steps I took round the playground”). This resulted in responses from 1087 school staff.

### 3.1. Respondent Demographics

Respondent demographics are presented in Table 1. Most respondents were female (*n* = 1020; 93.8%), aged 26- to 35-years (*n* = 382; 35.1%), white (*n* = 1025; 94.3%), and used a wearable for personal use (*n* = 816, 75.1%). Most respondents were classroom teachers (*n* = 697, 64.1%), had a professional degree (e.g., postgraduate certificate in education; PGCE) (*n* = 472; 43.4%) and worked in a state school (*n* = 630; 58%) in the United Kingdom (*n* = 921; 84.7%). Other countries included the United States of America (*n* = 107; 9.8%), Canada (*n* = 10; 0.9%), Australia (*n* = 7; 0.6%), New Zealand (*n* = 2; 0.2%), and countries in Europe (*n* = 22; 2%), Asia (*n* = 11; 1%) and Africa (*n* = 7; 0.6%). Most respondents taught or supported more than one age group.

### 3.2. Wearable Use: Prevalence and Characteristics

Most school staff had never implemented the use of wearables as teaching or support tools in school (*n* = 896; 82.4%), followed by currently using (*n* = 120; 11%) and previously used (*n* = 71; 6.5%) wearables as teaching or support tools. Table 2 displays the survey responses for the use of wearables as a teaching or support tool. Fitbits were the most used wearable brand, and most staff used wearables with students at least once a day (currently: 34.2%) or week (previously: 31%). Of the staff members who currently use a wearable with their students, most reported using the wearable as a teaching/support tool for more than 2-years (35.8%), but most staff who previously used a wearable with their students, used the device for less than 1-month (46.5%). Wearables were mostly implemented throughout the whole school day (currently: 50.8%, previously: 39.4%) and/or during physical education (P.E.) lessons (currently: 36.7%, previously: 40.8%). Steps were the most used feature, followed by heart rate or distance travelled, and use of the wearable’s partnering app or online dashboard ranged between 36.6% (previously) and 49.2% (currently). The wearable’s partnering app or online dashboard was most used with students in secondary school (currently: 54.2%, previously: 43.8%) than students in primary school (currently: 46.5%, previously: 36.5%).

The most common reasons for use were to promote student health and well-being, track student’s PA levels, and support student’s learning about health. Most school staff who stopped using a wearable as a teaching or support tool (previously used) reported devices broke/were lost (26.8%) or student’s lost interest in the devices (15.5%). The most common reason for non-use was cost (45.6%), followed by wearables being a distraction for students (40.8%) and fears students would break or lose the wearables (31.3%). Open-ended answers indicated some concerns around wearables’ impact on student’s mental health, well-being, or body image: “Could start an unhealthy obsession with tracking steps or calories at an impressionable age”, “Seems unnecessary and unfair, some children just aren’t as active or sporty as others. A tracker just gives them another goal to aim for that they might not meet. Enjoyment of physical activity is more important”.

### 3.3. Wearable Use: Content Analysis

The descriptive content analysis resulted in eight overarching themes and 24 sub-categories, which are presented in Supplementary Table S2, and are discussed in the following sections.

#### 3.3.1. How Wearables Are Used in Schools

Table 3 displays the results of the content analysis, which displays how school staff currently or have previously implemented the use of wearables as teaching or support tools in schools. Most staff used wearables to monitor student’s PA levels (currently: 38.8%, previously: 42.6%), increase student’s PA levels (currently: 44.8%, previously: 40.4%), via competitions, goals, and incentives, or to support student learning (currently: 23.9%, previously: 40.4%), such as maths and physics, human biology, and importance of PA for health.

#### 3.3.2. Future Wearable Use

Of the school staff who have previously (*n* = 71) or never (*n* = 896) implemented the use of wearables as a teaching or support tool, 66 (93%) and 624 (70%) were willing or very willing to use a wearable in the future. Table 4 displays the results of the content analysis, which shows how school staff would be willing to use wearables in the future. Most school staff were willing to use wearables for student educational purposes (previously: 54.5%, never: 46.5%), such as teaching students about the importance of PA on health. School staff were also willing to use wearables to monitor (previously: 40.9%, never: 35.7%) and increase (previously: 40.9%, never: 37.3%) student’s PA levels.

Despite willingness, some school staff (*n* = 27; 5.1%) reported concerns with students using wearables at school. Concerns were around increased staff workload and stress (“It would increase my workload and stress”), data privacy (“How will I use that information effectively without infringing on human rights”), and student mental health, with a focus on obsessive tracking (“If you could count calories and food intake, then this could contribute to obsessive eating habits”, “I would be willing but would be concerned with the welfare of the children with regard to them becoming obsessed with steps, calories”).

## 4. Discussion

This is the first study to explore child and adolescent widescale use of wearables, as implemented by school staff in schools, including why, how, and when they are used, and how school staff are willing to implement their use in the future. The findings from this study could inform future interventions and/or school-based policies surrounding the implementation of wearables within schools to promote PA or the learning about PA, in ways that are acceptable for school staff members.

Most school staff have never used wearables as a teaching or support tool in school (*n* = 896; 82.4%), followed by currently using (*n* = 120; 11%) and previously used (*n* = 71; 6.5%), with their students. When wearables were used, most school staff implemented them during all school hours (which included all classes and break time periods), or during physical education (P.E.) lessons, and used features ‘steps’, ‘heart rate’ and ‘distance travelled’. Previous research has explored the use of wearables during P.E. lessons and have reported wearables were useful tools for P.E. teachers to monitor and promote student’s PA levels [15,16]. Previous research has also found wearables can increase step counts during school breaktimes [10]. Considering ways to increase student’s PA levels, school staff reported using competitions and collective or individual goals. Previous research utilising wearables in intervention and feasibility studies have found that goal setting can increase children’s MVPA [10,23], however few studies have explored the impact of competition on PA [8]. Positively, most school staff who currently use wearables as a teaching or support tool have used the wearables, with their students, for over 2-years (35.8%), which demonstrates wearables long-term use. However, most school staff who have previously used wearables as a teaching or support tool, with their students, used the wearables for less than one-month (46.5%). Reasons for discontinuing use included the wearables becoming broken or lost (26.8%) or student’s losing interest in the wearables (15.5%). Therefore, if wearables are to be used as tools for promoting PA in schools, barriers to long-term use (e.g., maintaining student interest and maintenance of devices), and ways to encourage long-term use, must be considered.

It is recommended that PA should be incorporated throughout the whole school day, including academic lessons, P.E. and recess [17], with some countries recommending that children should achieve half of the recommended amount of daily MVPA (30-min) within the school environment [24]. Thus, it is promising that between 39.4% and 50.8% of school staff members reported their students’ used wearables throughout the whole school day. In particular, academic lessons are the least active part of a child’s day [3]. Few studies have explored the use of wearables during academic lessons, but those that have reported wearables’ acceptability [19] and ability to increase in student’s PA levels [25]. School staff, in the current sample, reported using wearables to monitor their student’s PA levels, which may inform their ability to adapt traditionally sedentary lessons to be more active. However, few school staff reported using wearables, or willingness to use wearables, to aid lesson planning, such as incorporating movement breaks into their lessons. Previous research has found that teachers prefer PA programs that can be sporadically implemented and in a short period of time [13], and movement breaks (brief intervals of PA) can increase children’s step-counts [26,27] and MVPA levels [27]. Not only that, a benefit of using wearables is that they automatically incorporate prompts or cues in the form of “reminders to move”, which provide regular reminders to be active, if periods of physical inactivity are detected [7,28]. Therefore, wearables can offer automated monitoring and prompts that can enable school staff to easily monitor their student’s real-time PA levels and incorporate movement breaks into academic lessons.

A common reason for not implementing the use of wearables as a teaching or support tool in schools was the expense of wearables (*n* = 409, 45.6%). A lack of financial support has previously been reported as a barrier of implementing PA programs by classroom teachers and headteachers [13]. Thus, the cost of wearables may be a barrier for most schools. Some schools (e.g., private schools) may have more financial resources to purchase wearables, and the potential for wearables to increase child health inequalities in PA levels based on financial support must be considered, in future research. Some countries, such as the United Kingdom (UK), offer government funding to improve PA and/or P.E. provisions, in primary schools (‘the P.E. and Sports Premium’) [29]. These initiatives may reduce the likelihood of wearables increasing health inequalities, but there must be sufficient evidence to support wearable’s ability to increase student’s PA levels before advising staff to use such funding on wearables. There is mixed evidence to suggest wearables, used within a school setting, can increase PA levels in 5- to 19-year-olds [12,30,31], and few studies have employed rigorous methods and study designs (e.g., randomised controlled trials) [32]. Therefore, further research is needed to support wearable’s actual ability to increase student PA levels to recommend wearables as a teaching or support tool. Fears that wearables could distract students from their schoolwork was also a common reason for school staff having never used a wearable as a teaching or support tool, with their students (*n* = 366, 40.8%). A previous feasibility study found that Fitbits initially distracted students from their classwork, but this did not continue past week one (out of a 12-week study) [33]. Therefore, concerns over the distractive nature of wearables may subside over time, and future studies may explore this further, by gaining in-depth qualitative accounts from school staff using wearables.

Some school staff also reported concerns over wearable’s impact on student mental health and well-being (e.g., obsessive tracking behaviours). Few studies have explored the impact of wearables on child and adolescent well-being, but one study reported that adolescents (14- 15-year-olds) improved their body satisfaction after using a Fitbit [12]. In adult wearable users, wearable use was negatively associated with psychological distress [34], and enhanced autonomy and perceived control over health and well-being [35]. Similarly, other studies have found that wearables (used as an intervention tool) can improve stress management and quality of life, in adults [36]. A minority of school staff expressed willingness to use wearables to increase, encourage or support other health behaviours, such as student well-being. Informing school staff about the best ways to use wearables to support a range of student health outcomes may aid the implementation of wearables in the school environment.

Despite potential barriers and concerns of using wearables, most school staff who have never or had previously used a wearable were willing to use wearables to monitor and increase their student’s PA levels and for student educational purposes. Potential uses for educational purposes included educating students about the importance of PA, incorporating wearables into maths, physics, and biology lessons to support the teaching of time, distance, statistics, and body functions. Indeed, the implementation of PA in schools is often overshadowed by teacher’s pressures to deliver traditional academic subjects [14], and previous research has found that teachers place importance on PA to positively impact student’s academic learning [13]. Thus, school staff’s acceptance of integrating wearables into academic lessons is promising. The concept of ‘embodied cognition’ provides insights into the advantages of incorporating movement within learning, emphasising the role of sensory and motor functions on cognition [37]. In such, learning through doing (being active) can inform educational concepts, such as maths. Previous research have also utilised wearables (e.g., Fitbit, BodyMedia) as part of maths lessons to teach geometry and statistics [25], and found that using wearables in this way can increase children’s PA levels [25] as well as their maths knowledge, such as data display and conceptions of statistics [38]. Thus, wearables have the potential to increase child and adolescent PA, as well as supporting student’s academic learning, which may overcome barriers of implementing PA initiatives in academic lessons (such as time and pressures to teach academic subjects).

### Strengths and Limitations

This is the first study to explore child and adolescent widescale use of wearables as implemented by school staff as teaching or support tools within schools. A strength of the current study includes the use of content analysis to quantify the large amounts of qualitative data into meaningful units that address the study’s research aims [21]. This provides an overview of the uses of wearables in schools and how future interventions and initiatives can employ wearable devices to promote PA awareness and PA levels in their students. Future research would benefit from employing in-depth qualitative methods to explore these findings further, particularly given some concerns around wearable use in schools (e.g., negative impact on student well-being). Likewise, future research can use the current study’s findings to formulate hypotheses and investigate causal mechanisms impacting the use of wearables in schools, and how they are used (e.g., similar research has explored what impacts wearable use in adults [39]. When doing so, future research should establish the validity and reliability of similar questionnaires. The large number of responses from classroom teachers (*n* = 697, 64.1%), rather than just focusing on school staff who teach P.E. lessons, is a strength of this survey. This provided insights into how wearables can be incorporated into periods of the school day that are typically inactive (e.g., academic lessons) and can be utilised for purposes beyond encouraging PA, such as student and staff educational purposes. The large sample size (*n* = 1087) and online distribution of this survey is also a strength. However, most respondents were female, of white ethnicity and lived in the United Kingdom. Although most school staff members, in the UK, are female and white [40], this is not representative of school staff around the world. Indeed, most published research, including intervention or feasibility studies using wearables to increase PA in children and adolescents [8], originates from higher income countries, such as the UK and USA [41]. Including responses from respondents from lower income and less Westernised countries, in the current study, provides initial steps into reducing such publication bias within the literature. However, with few responses from lower income countries, further research is needed to explore the use and barriers and facilitators of using wearables to promote PA in schools around the world, where school systems and resources may differ.

## 5. Conclusions

This is the first study to explore school staff’s habitual use and implementation of wearables as teaching or support tools to promote child or adolescent PA, in schools. Wearables are acceptable tools in the school environment, particularly when used to monitor and increase student PA levels or to educate students on the importance of PA or academic concepts (e.g., maths, physics, biology). This study demonstrates how wearables may be utilised across the school day, which may reduce periods of student physical inactivity, particularly during academic lessons [3]. By considering how school staff use, and are willing to use, wearables, researchers or health practitioners can consider how to appropriately integrate wearables into interventions or public health initiatives. The current study highlights when and how wearables can be used in schools, including the wearable features most used, and identifies key barriers, such as cost, concerns about wearables being distractions and having negative health impacts, of using wearables in schools. Future research may also benefit from collecting in-depth qualitative accounts from school staff members, providing further insights into the uses, barriers, and facilitators of using wearables in schools. From this, interventions or initiatives can be developed, where rigorous research can be conducted to empirically explore wearables impact on student’s PA levels, health, academic learning, and cognition.

## Figures and Tables

**Table 1:** Respondent demographics, *n* (%).

	Currently Use (*n* = 120)	Previously Used (*n* = 71)	Never Used (*n* = 896)	Total (*n* = 1087)
**Gender**				
Male	7 (5.8%)	4 (5.6%)	49 (5.5%)	60 (5.5%)
Female	112 (93.3%)	66 (93%)	842 (94%)	1020 (93.8%)
Non-binary	1 (0.8%)	1 (1.4%)	3 (0.3%)	5 (0.5%)
Rather not say	0	0	2 (0.2%)	2 (0.2%)
**Age**				
18–25 years	28 (23.3%)	7 (9.9%)	167 (18.6%)	202 (18.6%)
26–35 years	47 (39.2%)	21 (29.6%)	314 (35%)	382 (35.1%)
36–45 years	28 (23.3%)	30 (42.3%)	234 (26.1%)	292 (26.9%)
46–55 years	16 (13.3%)	13 (18.3%)	181 (20.2%)	210 (19.3%)
56–65 years	1 (1.2%)	0	0	1 (0.1%)
≥66 years	0	0	0	0
**Ethnicity**				
White	109 (90.8%)	67 (94.4%)	849 (94.8%)	1025 (94.3%)
Black	2 (1.7%)	0	7 (0.8%)	9 (0.8%)
Hispanic	4 (3.3%)	0	7 (0.8%)	11 (1%)
Asian	2 (1.7%)	2 (2.8%)	20 (2.2%)	24 (2.2%)
American Indian	1 (0.8%)	0	1 (0.1%)	2 (0.02%)
Pacific Islander	0	0	1 (0.1%)	1 (0.01%)
Mixed: Black and White	2 (1.7%)	2 (2.8%)	6 (0.7%)	10 (0.9%)
Mixed: Asian and White	0	0	5 (0.5%)	5 (0.5%)
**Education**				
<Undergraduate degree	18 (15%)	8 (11.3%)	103 (11.5%)	129 (11.9%)
Undergraduate degree	37 (30.8%)	19 (26.8%)	267 (29.8%)	323 (29.7%)
PGCE ^a^	53 (44.1%)	27 (38%)	392 (45.1%)	472 (43.4%)
Masters or PhD	12 (10%)	17 (23.9%)	134 (15%)	163 (15%)
**Type of school**				
State	78 (65%)	41 (57.7%)	511 (57%)	630 (58%)
Private	8 (6.7%)	8 (11.3%)	59 (6.7%)	75 (6.9%)
Academy	23 (19.2%)	16 (22.5%)	216 (24.1%)	255 (23.5%)
Special educational needs (SEN) ^b^	2 (1.7%)	2 (2.8%)	46 (5.1%)	50 (4.6%)
Faith	9 (7.5%)	4 (5.6%)	64 (7.1%)	77 (7.1%)
Other	0	0	0	0
**Job role**				
Classroom teacher	66 (55%)	38 (53.5%)	593 (66.3%)	697 (64.1%)
Physical education (P.E.) teacher	23 (19.2%)	23 (32.4%)	58 (6.5%)	104 (9.6%)
Teaching assistant	24 (20%)	7 (9.9%)	158 (17.6%)	189 (17.4%)
Headteacher	0	0	8 (0.9%)	8 (0.7%)
Deputy headteacher	0	1 (1.4%)	25 (2.8%)	26 (2.4%)
Trainee teacher	0	0	0	0
SEN ^b^ lead or support worker	3 (2.6%)	0	33 (3.7%)	36 (3.3%)
Substitute teacher	0	0	3 (0.3%)	3 (0.2%)
Leadership staff (e.g., head of year)	1 (0.8%)	1 (1.4%)	7 (0.8%)	9 (0.8%)
Midday staff	0	0	2 (0.2%)	2 (0.2%)
Admin or support role	1 (0.8%)	0	3 (0.3%)	4 (0.4%)
Librarian	1 (0.8%)	1 (1.4%)	0	2 (0.2%)
Forest school lead	0	0	4 (0.4%)	4 (0.4%)
Other	1 (0.8%)	0	2 (0.2%)	3 (0.2%)
**Age group teach/support ^c^**				
5-years	26 (21.7%)	19 (26.8%)	233 (26%)	278 (25.6%)
6-years	35 (29.2%)	21 (29.6%)	275 (30.7%)	331 (30.5%)
7-years	53 (44.2%)	29 (40.8%)	355 (39.6%)	437 (40.20%)
8-years	57 (47.5%)	30 (42.3%)	381 (42.5%)	468 (43.1%)
9-years	53 (44.2%)	31 (43.7%)	269 (41.2%)	353 (32.5%)
10-years	45 (37.5%)	31 (43.7%)	331 (36.9%)	407 (37.4%)
11-years	49 (40.8%)	38 (53.5%)	337 (37.6%)	424 (39%)
12-years	28 (23.3%)	18 (25.4%)	171 (19.1%)	217 (20%)
13-years	28 (23.3%)	16 (22.5%)	164 (18.3%)	425 (39.1%)
14-years	26 (21.7%)	14 (19.7%)	164 (18.3%)	204 (18.8%)
15-years	20 (16.7%)	14 (19.7%)	158 (17.6%)	192 (17.7%)
16-years	19 (15.8%)	14 (19.7%)	154 (17.2%)	187 (17.2%)
**Personal wearable use**				
Currently using	117 (97.5%)	46 (64.8%)	653 (72.9%)	816 (75.1%)
Previously used	3 (2.5%)	23 (32.4%)	131 (14.6%)	157 (14.4%)
Never used	0	2 (2.8%)	112 (12.5%)	114 (10.5%)

^a^ Postgraduate certificate in education. ^b^ Special educational needs. ^c^ Respondents could select more than one answer.

**Table 2:** School staff’s use of wearables as teaching or support tools in school, *n* (%).

	Currently Use (*n* = 120)	Previously Used (*n* = 71)	Never Used (*n* = 896)
**Wearable brand ^a^**			
Fitbit	43 (35.8%)	31 (2.9%)	n/a
Garmin	17 (14.2%)	10 (0.9%)	
Misfit	0	1 (0.1%)	
Apple	55 (45.8%)	7 (0.6%)	
Samsung	12 (10%)	6 (0.6%)	
Huawei	2 (1.7%)	1 (0.1%)	
Moki	7 (5.8%)	5 (0.5%)	
Amazon	6 (5%)	4 (0.4%)	
Xiaomi	2 (1.7%)	0	
Unbranded	2 (1.7%)	8 (0.7%)	
Unsure	0	5 (0.5%)	
Other	4 (3.3%)	5 (0.5%)	
Use(d) more than one device:	30 (25%)	12 (16.9%)	
**Frequency of use**			
Multiple times a day	26 (21.7%)	7 (9.9%)	n/a
At least once a day	41 (34.2%)	17 (23.9%)	
At least once a week	31 (25.8%)	22 (31%)	
At least once a month	12 (10%)	6 (8.5%)	
At least once a year	7 (5.8%)	11 (15.5%)	
Less than once a year	3 (2.5%)	8 (11.3%)	
Unsure	0	0	
**Duration of use**			
<1 month	7 (5.8%)	33 (46.5%)	n/a
1–5 months	23 (19.2%)	20 (28.2%)	
6–11 months	18 (15%)	8 (11.3%)	
1–2 years	29 (24.2%)	7 (9.9%)	
>2 years	43 (35.8%)	3 (4.2%)	
Unsure	0	0	
**Stopped using**			
<1 month ago	n/a	8 (11.3%)	n/a
1–5 months ago		16 (22.5%)	
6–11 months ago		10 (14.1%)	
1–2 years ago		23 (32.4%)	
>2 years ago		14 (19.7%)	
Unsure		0	
**When used ^a^**			
All school hours	61 (50.8%)	28 (39.4%)	n/a
Physical education (P.E.) lessons	44 (36.7%)	29 (40.8%)	
Recess/break periods	19 (15.8%)	12 (16.9%)	
Core lessons (e.g., English, Math, Science)	19 (15.8%)	8 (11.3%)	
Other lessons (e.g., Art, Language, IT)	4 (3.3%)	1 (1.4%)	
Daily mile	1 (0.8%)	3 (4%)	
All day (incl. non-school hours)	1 (0.8%)	0	
Did not provide an answer	1 (0.8%)	2 (2.8%)	
**Feature(s) used ^a^**			
Steps	82 (68.3%)	53 (74.7%)	n/a
Heart rate	66 (55%)	39 (54.9%)	
Calories burned/expended	40 (33.3%)	9 (12.7%)	
Active/intensity/zone minutes	28 (23.3%)	16 (22.5%)	
Distance/miles/km travelled	66 (55%)	17 (23.9%)	
Stairs/floors climbed	19 (15.8%)	3 (4%)	
Sleep tracking	13 (10.8%)	4 (5.6%)	
Virtual rewards/trophies	8 (6.7%)	1 (1.4%)	
PA challenges	34 (28.3%)	5 (7%)	
Social media/community components	6 (5%)	1 (1.4%)	
Food intake	3 (2.5%)	1 (1.4%)	
Water intake	10 (8.3%)	2 (2.8%)	
Weight status/change	7 (5.8%)	1 (1.4%)	
Unsure	0	0	
Other:			
Timer/stopwatch	23 (19.2%)	0	
**Use partnering app/online dashboard**			
Yes	59 (49.2%)	26 (36.6%)	
No	53 (44.2%)	42 (59.2%)	
Unsure	8 (6.6%)	3 (4.2%)	
**Reason for use ^a^**			
Interest in new technology	39 (32.5%)	10 (14.1%)	n/a
Promote student’s health/well-being	61 (50.8%)	33 (46.5%)	
Required/encouraged by senior staff	7 (5.8%)	5 (7%)	
Track student’s PA	40 (33.3%)	33 (46.5%)	
Support student’s learning about health	41 (34.2%)	27 (38%)	
Support student’s academic learning	31 (25.8%)	12 (16.9%)	
Increase student’s PA	40 (33.3%)	21 (29.6%)	
Track student’s health unrelated to PA	15 (12.5%)	7 (8.9%)	
Stopwatch/timer	13 (10.8%)	1 (1.4%)	
Study	0	2 (2.8%)	
**Reason for stopping using ^a^**			
Too expensive	n/a	5 (7%)	n/a
Lost interest		11 (15.5%)	
Devices broke or lost		19 (26.8%)	
Senior staff did not support		2 (2.8%)	
Students did not enjoy		5 (7%)	
Distraction from schoolwork		8 (11.3%)	
Students did not understand how to use		2 (2.8%)	
Students’ parents were not supportive		1 (1.4%)	
Study ended		8 (11.3%)	
COVID-19		8 (11.3%)	
Technical issues/burden		4 (5.6%)	
Negative health outcomes		3 (4.2%)	
Other (e.g., removal of watches during P.E., left school, curriculum change)		9 (12.7%)	
**Reason for not using ^a^**			
Too expensive	n/a	n/a	409 (45.6%)
No interest in using			154 (17.2%)
Fears of losing/breaking devices			280 (31.3%)
Students would not enjoy			25 (2.8%)
Senior staff would not support			98 (10.9%)
Fears they would distract students			366 (40.8%)
Students would not understand how to use wearable			133 (14.8%)
Parents would not support			106 (11.8%)
Other:			
Not considered			17 (1.9%)
Fears of negative health outcomes			30 (3.3%)
Unsure how they could be used			21 (2.3%)
Not relevant/useful			7 (0.8%)
Safety concerns			25 (2.8%)
No access			3 (0.3%)
No need			32 (3.6%)

^a^ Respondents could select more than one answer.

**Table 3:** How school staff implement the use of wearables in schools, *n* (%).

	Currently Use	Previously Used	Example Quotations
	(*n* = 120)	(*n* = 71)	
**Excluded responses**	**53 (44.2%)**	**24 (33.8%)**	“I really use it for myself” “During P.E. lessons”
No answer	7 (5.8%)	9 (12.7%)	
Does not address ‘how’	10 (8.3%)	11 (15.5%)	
Students were not wearable users	36 (3%)	4 (5.6%)	
	*n* = 67	*n* = 47	
**Monitor PA or increase awareness of PA levels ^a^**	**26 (38.8%)**	**20 (42.6%)**	“We chartered their steps” “Each child had a journal to record their steps” “Allow students and parents to better understand how much exercise they are doing each day”
Teacher/staff monitors students PA	5 (7.5%)	5 (10.6%)	
Student monitors own PA	4 (6%)	4 (8.5%)	
Unspecified monitoring	17 (25.4%)	13 (27.7%)	
Other: Encourage parents to monitor	1 (1.5%)	0	
**Monitor other behaviour ^a^**	**2 (3%)**	**0**	“Track their physical activity, sleep and water intake”
Water intake	1 (1.5%)	0	
Sleep	1 (1.5%)	0	
Food intake	1 (1.5%)	0	
**Comparison of physical activity levels ^a^**	**9 (13.4%)**	**8 (17%)**	“I compare steps with students” “Children compared heart rates”
Teacher-student comparison	4 (6%)	2 (4.3%)	
Between-student comparison	0	3 (6.4%)	
Within-student comparison	1 (1.5%)	0	
Unspecified comparison	4 (6%)	4 (8.5%)	
Other	0	0	
**Increase PA ^a^**	**30 (44.8%)**	**19 (40.4%)**	“Step competitions between classes” “Keep heart rate in target zones” “Winning class each week got extra reward time”
Teacher-student competition	3 (4.5%)	0	
Student-student competition	1 (1.5%)	0	
Class-class/team-team competition	3 (4.5%)	4 (8.5%)	
School-school competition	1 (1.5%)	0	
Unspecified competition	5 (7.5%)	0	
Individual goals	6 (9%)	2 (4.3%)	
Collective goals	6 (9%)	4 (8.5%)	
Unspecified goals	3 (4.5%)	1 (2.1%)	
Rewards or incentives	1 (1.5%)	3 (6.4%)	
Other	0	0	
**Increase or support other health behaviour(s) ^a^**	**0**	**1 (2.1%)**	“Promote well-being”
Well-being/mental health	0	1 (2.1%)	
**Student educational purposes ^a^**	**16 (23.9%)**	**19 (40.4%)**	Recorded the steps and used it in a maths and science to make graphs and analyse the data” “We discuss why moving benefits health and wellbeing” “Compare heart rate when children have measured theirs manually”
Maths and physics (e.g., time, distance, statistics)	5 (7.5%)	6 (12.8%)	
Human biology (e.g., body functions)	7 (10.5%)	9 (19.1%)	
Importance of PA for health	5 (7.5%)	7 (14.9%)	
Other: GPS	1 (1.5%)	0	
Other: Education around wearable accuracy	1 (1.5%)	1 (2.1%)	
**Staff educational purposes ^a^**	**4 (6%)**	**3 (6.4%)**	“To support teachers to understand why they should include PA in lessons” “To measure the impact of our PESSPA curriculum. This supports the planning of all P.E. lessons”
Lesson planning (incl. movement breaks)	2 (3%)	3 (6.4%)	
Impact of curriculum on PA levels	3 (4.5%)	1 (2.1%)	
Increase other staff’s knowledge to inform lesson plans	0	1 (2.1%)	
**Other ^a^**	**2 (3%)**	**6 (12.8%)**	“Encourage students to take responsibility for their health” “A targeted group used them and once monitored they fed back to school council”
Encourage students to take ownership of own health	1 (1.5%)	0	
Measure of PA	1 (1.5%)	2 (4.3%)	
Measure impact of existing project/research study	0	3 (6.4%)	
Feedback to school council	0	1 (2.1%)	

^a^ Responses could be categorised into more than one category, including sub-categories. Percentages calculated using the total number of valid responses (currently uses: *n* = 67, previously used: *n* = 47). **Bold:** overarching themes.

**Table 4:** How school staff are willing to implement the use of wearables in schools, *n* (%).

	Previously Used (*n* = 66)	Never Used (*n* = 624)	Example Quotations
**Excluded responses**	**22 (33.3%)**	**134 (21.5%)**	“As part of a rowing scheme” “Easy to use, will do again”
No answer	5 (7.6%)	50 (8%)	
Does not address ‘how’	13 (19.7%)	21 (3.4%)	
Students were not wearable users	4 (6.1%)	63 (10.1%)	
	*n* = 44	*n* = 490	
**Monitor PA or increase awareness of PA levels ^a^**	**18 (40.9%)**	**175 (35.7%)**	“Have students monitor their heart rate during class activities” “It’d be great to use trackers to measure steps at home”
Teacher/staff monitors students PA	3 (6.8%)	35 (7.1%)	
Student monitors own PA	6 (13.6%)	60 (12.2%)	
Unspecified monitoring	9 (20.5%)	77 (15.7%)	
Other: Encourage parents to monitor	0	3 (0.6%)	
**Monitor other behaviour ^a^**	**0**	**29 (5.9%)**	“Monitoring water intake” “It would be interesting to see students sleep patterns”
Water intake	0	10 (2%)	
Sleep	0	14 (2.9%)	
Food intake	0	3 (0.6%)	
Overall health and lifestyle	0	1 (0.2%)	
Mental health/well-being	0	2 (0.4%)	
**Comparison of physical activity levels ^a^**	**2 (4.5%)**	**19 (3.9%)**	“Interesting to compare the teacher and student steps” “To see how much physical activity students complete in relation to guidelines”
Teacher-student comparison	0	2 (0.4%)	
Between-student comparison	1 (2.3%)	2 (0.4%)	
Within-student comparison	0	4 (0.8%)	
Unspecified comparison	0	9 (1.8%)	
Other: Comparison to guidelines	1 (2.3%)	5 (1%)	
**Other comparisons ^a^**	**0**	**1 (0.2%)**	“Monitoring water intake… Using this data as a comparative to peers”
Between-student water intake	0	1 (0.2%)	
**Increase PA ^a^**	**18 (40.9%)**	**183 (37.3%)**	“Try to stay within target zones” “Team competitions” “To have some kind of a steps challenge” “Check their average minutes to try and go for 30 min every day”
Teacher-student competition	0	1 (0.2%)	
Student-student competition	1 (2.3%)	14 (2.9%)	
Class-class/team-team competition	0	5 (1%)	
School-school competition	0	0	
Unspecified competition	1 (2.3%)	27 (5.5%)	
Individual goals	1 (2.3%)	19 (3.9%)	
Collective goals	3 (6.8%)	4 (0.8%)	
Unspecified goals	1 (2.3%)	18 (3.7%)	
Rewards or incentives	1 (2.3%)	6 (1.2%)	
Other: Via device features (feedback, reminders to move)	0	4 (0.8%)	
Other: Increase PA outside of school hours	0	5 (1%)	
**Increase or support other health behaviour(s) ^a^**	**2 (4.5%)**	**21 (4.3%)**	“To promote mental well-being” “Water intake, set a reasonable goal of how much we should be drinking in a day to stay hydrated” “Improving sleep/rest at home” “Stress relief strategies”
Well-being/mental health	1 (2.3%)	12 (2.4%)	
Overall health and lifestyle	1 (2.3%)	3 (0.6%)	
Sleep	0	3 (0.6%)	
Water intake	0	6 (1.2%)	
Learning	0	1 (0.2%)	
Stress management	0	1 (0.2%)	
**Student educational purposes ^a^**	**24 (54.5%)**	**228 (46.5%)**	“I would like to show the children how to monitor their heart rate and why it’s important” “To support children’s understanding of their own health linked to science lessons” “I would have students observe the colour of the light on the back of the device based on our discussion of spectroscopy”
Maths and physics (e.g., time, distance, statistics)	10 (22.7%)	75 (15.3%)	
Human biology (e.g., body functions)	5 (11.4%)	60 (12.2%)	
Importance of PA for health	12 (27.3%)	122 (24.9%)	
Other: importance of other behaviours (sleep, water intake)	0	19 (3.9%)	
Other: GPS, geography (maps)	1 (2.3%)	2 (0.4%)	
Other: Education around wearable features and accuracy	2 (4.5%)	5 (1%)	
Other: Reading and literacy	0	5 (1%)	
Other: Telling the time	1 (2.3%)	2 (0.4%)	
**Staff educational purposes ^a^**	**1 (2.3%)**	**25 (5.1%)**	“Having an activity tracker would mean reminders for regular movement breaks” “I can understand why they might be underachieving, e.g., if they’ve had less sleep”
Lesson planning (incl. movement breaks)	1 (2.3%)	12 (2.4%)	
Impact of curriculum on PA levels	0	1 (0.2%)	
Increase other staff’s knowledge to inform lesson plans	0	1 (0.2%)	
Using outputs to understand student behaviour	0	11 (2.2%)	
**Other ^a^**	**2 (4.5%)**	**15 (3%)**	“A good way to make children aware of their health and to take control of it” “I think that it would be good to collect the data on the children instead of doing other types of assessment on their fitness levels”
Encourage students to take ownership of own health	0	11 (2.2%)	
Measure of PA	0	3 (0.6%)	
Measure impact of existing project/research study	1 (2.3%)	0	
Required for course or curriculum	1 (2.3%)	0	
Trial prior to using	0	1 (0.2%)	

^a^ Responses could be categorised into more than one category, including sub-categories. Percentages calculated using the total number of valid responses (previously used: *n* = 44, never used: *n* = 490). **Bold:** overarching themes.

## Data Availability

The data presented in this study are available on request from the corresponding author.

## Supplementary Materials

The following supporting information can be downloaded at: https://www.mdpi.com/article/10.3390/ijerph192114067/s1. Supplementary Table S1: Survey questions and response options; Supplementary Table S2: Developed themes and sub-categories for the content analysis.
