# Supplementary material for: The Use of Wearable Activity Trackers in Schools to Promote Child and Adolescent Physical Activity: A Descriptive Content Analysis of School Staff’s Perspectives
Source: Int J Environ Res Public Health. 2022 Oct 28;19(21):14067. doi: 10.3390/ijerph192114067 (PMC9654652; doi:10.3390/ijerph192114067)
Supplement: Supplementary file 1 [file ijerph-19-14067-s001.zip › ijerph-1970520-supplementary.pdf]

The use of wearable activity trackers in schools to promote physical activity: A content analysis

Supplementary Materials

Supplementary Table S1. Survey questions and response options.

	Question	Responses
Demographic data (respondent)	o What is your Country of Residence?<br>o What gender do you identify with?<br>o What is your age?<br>o What is your ethnicity?<br>o What is your highest educational qualification?<br>o Do you use, or have you previously used a wearable activity/fitness tracker (e.g., Fitbit, Garmin, Misfit, Apple)?	o Australia, Canada, New Zealand, United Kingdom, United States of America, Other: please state<br>o Male, Female, Non-binary, Rather not say, Other: please state.<br>o 18-25 years, 26-35 years, 36-45 years, 46-55 years, 56-65 years, 66 years or over.<br>o White (e.g., English, Irish, Scottish, German), Black (e.g., African, Caribbean), Hispanic, Latino or Spanish original (e.g., Mexican, Cuban, Puerto Rican), Asian (e.g. Indian, Pakistani, Bangladeshi, Chinese, Korean), American Indian or Alaska Native (e.g. Navajo nation, Blackfeet tribe), Middle Eastern or North African (e.g. Egyptian, Syrian, Moroccan), Native Hawaiian or other Pacific Islanders (e.g. Fijian, Samoan), Mixed: White and Black Caribbean, Mixed: White and Black African, Mixed: White and Asian, Other: please state.<br>o No schooling, Primary education, Secondary education, Advanced subsidiary level, Advanced level, Undergraduate degree, Master's degree, Professional degree, Doctoral degree (or equivalent for all), Other: please state.<br>o I currently use a wearable activity/fitness tracker, I have previously (but no longer) used a wearable activity/fitness tracker, I have never used a wearable activity/fitness tracker
Demographic data (school, job role, and students)	o What type of school do you work in?<br>o What best describes your job role?<br>o What age group do you teach/support? Please select all that apply	o State, Private, Academy, Special Educational Needs (SEN) school, Faith school, Other: please state.<br>o Classroom teacher, Physical education (P.E) teacher, Teaching assistant, Headteacher, Deputy headteacher, Trainee teacher, Special education needs (SEN) lead/support worker, Other: please state.

		o 5 years, 6 years, 7 years, 8 years, 9 years, 10 years, 11 years, 12 years, 13 years, 14 years, 15 years, 16 years.
Wearable use (prevalence)	o Do you currently use, or have you previously used, wearable activity/fitness trackers as a teaching/support tool?	o I currently use a wearable activity/fitness tracker as a teaching/support tool, I have previously (but no longer) used a wearable activity/fitness tracker as a teaching/support tool, I have never used a wearable activity/fitness tracker as a teaching/support tool.
Wearable use (characteristics)	**Currently using/previously used:**<br>o What brand of wearable activity/fitness tracker(s) do you use as a teaching/support tool? Please select all that apply<br>o How long have/did you use a wearable activity/fitness tracker as a teaching/support tool?<br>o On average, how often do/did you use a wearable activity/fitness tracker as a teaching/support tool?<br><br>**Previously used:**<br>o When did you stop using wearable activity/fitness trackers as a teaching/support tool for the pupils/students you work with?	o Fitbit, Garmin, Misfit, Apple, Samsung, Huawei, Moki, Amazon, Xiaomi, Other: please state.<br>o Less than 1 month, 1-5 months, 6-11 months, 1 year – 2 years, More than 2 years, Unsure.<br>o Multiple times a day, At least once a day, At least once a week, At least once a month, At least once a year, Less than once a year, Unsure.<br><br>o Less than 1 month ago, 1–5 months ago, 6–11 months ago, 1 year–2 years ago, More than 2 years ago
Wearable use (reasons for use/non-use)	**Currently using/previously used:**<br>o What is the reason(s) for you using wearable activity/fitness trackers as a teaching/support tool for the pupils/students you work with? Please select all that apply<br><br>**Previously used:**	o Interest in new technology, Promote pupil/student health and wellbeing, Required/encouraged to by senior school staff members, Track/monitor pupils/students' physical activity, Support pupils/students learning about health, Support pupils/students learning in academic subjects (e.g., mathematics), Increase pupils/students' physical activity, Track/monitor pupils/students' health, Other. Please state:<br>o Too expensive, Pupils/students lost interest in the devices, Devices were lost or broken, Safeguarding issues around using the devices in school, The school I work in lost interest in using wearable activity/fitness trackers, The devices did not increase

	○ What was the reason(s) for you stopping using a wearable activity/fitness tracker as a teaching/support tool? Please select all that apply<br><br>**Never used:**<br>○ What is the reason(s) for you not using a wearable activity/fitness tracker as a teaching/support tool? Please select all that apply	pupils/students physical activity levels, The school's senior staff members no longer supported the use of wearable activity/fitness trackers in school , Pupils/students did not enjoy using the devices, I, or the school I work in, used other ways to increase pupils/students' physical activity, I, or the school I work in, used other ways to monitor/track pupils/students' physical activity, Devices distracted pupils/students from their school work, Pupils/students did not understand how to use them, Parents/guardians of the pupils/students did not support the use of wearable activity/fitness trackers at school, Other: please state.<br>○ Too expensive, Pupils/students have no interest in using wearable activity/fitness trackers, Fears/concerns the devices would get lost or broken, Safeguarding fears/concerns around using such devices in school, The school I work in has no interest in using wearable activity/fitness trackers, Pupils/students would not enjoy using wearable activity/fitness trackers, I do not believe wearable activity/fitness trackers can increase pupils/students physical activity levels, The school's senior staff members do not support the use of wearable activity/fitness trackers in school, I, or the school I work in, use other ways to increase pupil/student physical activity, I, or the school I work in, use other ways to track/monitor pupil/student physical activity, Fears/concerns the devices would distract pupils/students from their school work, Pupils/students would not understand how to use a wearable activity/fitness tracker, Parents/guardian of the pupils/students would not support the use of wearable activity/fitness trackers at school, Other: please state.
Wearable use (when used)	**Currently using/previously used:**<br>○ When do you use wearable activity/fitness trackers as a teaching/support tool for the pupils/students you work with?	○ All school hours, P.E. lessons, Recess/breaktime/lunch periods, During core lessons (English, Maths and Science), During other lessons (Art, Language, IT), Other: please state.

Wearable use (how used)	**Currently using/previously used:**<br>o What features of the wearable activity/fitness tracker do/did you use as a teaching/support tool?<br>o Do/did you use the wearable activity/fitness tracker's partnering app/online dashboard as a teaching/support tool?<br>o Please explain how you use wearable activity/fitness trackers as a teaching/support tool	o Steps, Heart rate, Calories burned/expended, Active/intensity/zone minutes, Distance/miles/km travelled, Stairs/floors climbed, Sleep tracking, Virtual rewards/badges/trophies, Physical activity challenges/competitions, Social media/community components, Water intake, Food intake, Weight loss, Other: Please state.<br>o Yes, No, Unsure<br>o Open ended question.
Wearable (willingness to use)	**Never and previously used:**<br>o If you were given the opportunity, how willing would you be to use wearable activity/fitness trackers as a teaching/support tool **at school**, in the future?<br>o Please explain how you would be willing to use wearable activity/fitness trackers as a teaching/support tool	o Very willing, Willing, Unwilling, Very unwilling, Unsure<br>o Open ended question.

Supplementary Table S2. Developed themes and sub-categories for the content analysis.

Overarching theme	Sub-category
**Wearable is used to monitor PA or increase awareness of PA levels**	Teacher/staff monitors students PA<br>Student monitors own PA<br>Unclear who is monitoring students PA<br>Other – indicate
**Monitor other behaviour(s)** – indicate behaviour and who is monitoring	N/A
**PA comparison**	Teacher-student comparison<br>Between-student comparison<br>Within-student comparison<br>Comparison unclear<br>Other – indicate comparison
**Increase PA**	Teacher-student competition<br>Student-student competition<br>Class-class/team-team competition<br>School-school competition<br>Competition – unclear who between<br>Individual goals<br>Collective goals<br>Goals - unclear<br>Rewards or incentives<br>Other – indicate
**Increase or support other health behaviour(s)** – indicate health behaviour and how	N/A
**Student educational purposes**	Maths and physics (e.g., time, distance, statistics)<br>Human biology (e.g., body functions)<br>Importance of PA for health<br>Other –indicate
**Staff educational purposes** – indicate how	N/A
**Other** - indicate	N/A
